# Local and systemic effects in e-cigarette users compared to cigarette smokers, dual users, and non-smokers

**DOI:** 10.1186/s12931-025-03289-4

**Published:** 2025-06-04

**Authors:** Shanzina Iasmin Sompa, Jie Ji, Mizanur Rahman, Bengt Sjögren, Swapna Upadhyay, Koustav Ganguly, Anna-Carin Olin, Anna Bergström, Lena Palmberg

**Affiliations:** 1 Integrative Toxicology unit, Institute of Environmental Medicine, Karolinska Institutet, Stockholm, 17177 Sweden; 2 School of Public Health and Community Medicine, Gothenburg University, Gothenburg, Sweden

**Keywords:** E-cigarettes, Dual use, FeNO, TLR, Cytokines

## Abstract

**Background:**

The use of electronic (e)-cigarettes in the long term has been associated with an increased risk of respiratory diseases. Dual use of e-cigarettes and traditional cigarettes may increase these risks even more due to the combined exposure effects of these products. The aim of this study was to investigate the local and systemic effects of e-cigarette use for more than one year and compare them with healthy non-smokers, cigarette smokers, and dual users.

**Methods:**

The clinical study was conducted among 22 healthy non-smokers, 20 e-cigarette users, 20 cigarette smokers, and 20 dual users. Participants were matched with age and BMI, had normal baseline lung function, and had no allergies. Exhaled FeNO and bronchial responsiveness were assessed along with reactive oxygen species (ROS), toll-like receptor (TLR) expression, and inflammatory cytokines in blood and sputum.

**Results:**

Exhaled FeNO was higher in e-cigarette users (14 ppb, *p* = 0.04) and lower in cigarette smokers (9 ppb, *p* = 0.04) compared to healthy non-smokers (11 ppb). Bronchial responsiveness was increased in e-cigarette users (1.9 mg, *p* = 0.01) and cigarette smokers (1.9 mg, *p* = 0.01) compared to healthy non-smokers (2.9 mg). ROS in blood and sputum in e-cigarette users (*p* = 0.005 and *p* = 0.04) and dual users (*p* = 0.003 and *p* = 0.04) were increased. Also, TLR2 expression in blood granulocytes in all exposed groups (*p* = 0.001), TLR2 and TLR4 expression in sputum in e-cigarette users (*p* = 0.04 and *p* = 0.03) and dual users (*p* < 0.0001 and *p* = 0.004) were increased. Moreover, the percentage of IL13 and IFNγ cytokine-producing T cells in blood were increased in e-cigarette users (*p* = 0.0001 and *p* < 0.0001) and dual users (*p* = 0.001 and *p* < 0.0001).

**Conclusion:**

Our research indicates that both local and systemic inflammatory responses, along with innate immune receptor activity, were significantly altered in e-cigarette users and dual users. Notably, these alterations were detected in e-cigarette users within a short timeframe of just 1 to 3 years of use.

**Clinical trial number:**

Not applicable.

**Supplementary Information:**

The online version contains supplementary material available at 10.1186/s12931-025-03289-4.

## Introduction

For decades, cigarette smoking has been a well-known risk factor for impaired health including pulmonary diseases such as chronic obstructive pulmonary disease (COPD), bronchitis, and lung cancer [1, 2]. As an alternative, non-combustible electronic (e)-cigarettes were invented which heat e-liquid and deliver an aerosol for inhalation [3]. Since 2014, e-cigarettes have emerged as a public health problem, with over 2.5 million current users among youth in the U.S., making them the most common electronic nicotine delivery system and alternative tobacco product [4].

Recent scientific evidence has found the association of e-cigarette use with significant lung injury and respiratory diseases that are comparable to those caused by traditional cigarettes [5–8]. In addition, the use of e-cigarettes can contribute to the development of cardiovascular and other systemic diseases [8]. A major concern about e-cigarette use is the potential health risks associated with nicotine and other toxic chemicals found in the aerosol like fine/ultrafine particles, metals, carcinogens, additives, and flavourings compounds [9]. Furthermore, nicotine-containing e-cigarettes can be a gateway to traditional cigarettes or other tobacco products such as snus or waterpipes [10, 11].

Flavouring chemical diacetyl, present in many e-liquids, has been reported to be associated with irreversible lung disease bronchiolitis obliterans [12]. In our previous study, there was a significantly higher prevalence of chronic bronchitis-like symptoms, such as cough and mucous production, among e-cigarette users compared to non-smokers [13]. Several epidemiological studies have also found the association of e-cigarette use with increased prevalence of cough, phlegm, and breathing difficulty [8, 14–20]. Other studies have found the association of e-cigarette use with self-reported increased exacerbation of asthma or chronic bronchitis symptoms, and airway irritation [8, 21–23], impaired lung function, altered Fraction of exhaled Nitric Oxide (FeNO) level, and increased airway reactivity [24–28].

The emergence of e-cigarettes or vaping-associated lung diseases (EVALI) in 2019 affected thousands of e-cigarette users, leading to hospitalizations and several deaths due to severe lung injury [7]. Investigating the systemic effects of e-cigarette use has become even more relevant since then. Experimental studies have consistently found that e-cigarettes may impair immune function in the lungs, disrupt mucociliary function, increase susceptibility to infection, and alter systemic biomarkers related to chronic lung diseases [6, 29]. Several studies have found e-cigarettes to cause cytotoxicity, inflammation, oxidative stress, DNA damage, and cell death, the common biological processes relevant to respiratory diseases [23, 29–33]. Interestingly, many of these effects occur independently of nicotine content, even with basic e-cigarette components like propylene glycol and glycerol exhibiting cytotoxic effects on lung cells [23, 34].

A common misconception among cigarette smokers is that e-cigarettes may deliver fewer toxic chemicals than traditional cigarettes, leading many to transition to e-cigarettes as a safer alternative or as a tool to quit smoking [6, 35, 36], although the role of e-cigarettes in reducing harm still lacks strong scientific evidence [6, 37]. In addition, most smokers find it challenging to switch over to e-cigarettes or quit cigarette smoking successfully and instead continue to use both products simultaneously (dual use) [38–40]. Currently, the knowledge of the long-term health effects of dual use, particularly regarding lung health, is insufficient. The level of nicotine intake may be higher among dual users compared to single tobacco product users [16, 41]. The combined exposure to toxic chemicals, including nicotine released from both e-cigarettes and traditional cigarettes can elevate the risk of developing addiction, heart diseases, stroke, respiratory problems like airway irritation, cough, breathing difficulty, and chronic bronchitis [16, 42]. In addition, dual use can expose consumers to a combination of health risks that are yet to evidence [40].

Given that, e-cigarettes are still relatively new, more comprehensive research is needed to fully understand the mechanism behind the health effects caused by e-cigarette use or dual use [20]. Therefore, the present study aimed to investigate the long-term local and systemic effects of e-cigarette use in comparison to cigarette smokers, dual users, and healthy non-smokers. Local effects in the airways were assessed by measuring FeNO and bronchial hyperresponsiveness. In addition, local effects regarding oxidative stress, surface expression of TLR, and cytokines release were measured in sputum and saliva, while systemic effects were measured in blood/serum. We hypothesized that e-cigarettes alone could cause harmful effects similar to or even more detrimental than combustible cigarettes. We also hypothesized that e-cigarettes may enhance these harmful effects among dual users.

## Materials and methods

### Study participants and study design

All participants were recruited through advertisement, and the study was conducted between 2019 and April 2024. The study was postponed from April 2020 to October 2021 due to COVID-19. Vaccination against COVID-19 was demanded for participation from October 2021 onwards.

A total of 82 participants were categorized into four groups, i.e., 22 healthy non-smokers (who never smoked any tobacco products), 20 e-cigarette users, 20 cigarette smokers, and 20 dual users (who used both e-cigarettes and traditional cigarettes with/without the use of snus). The eligible age to participate in the study was between 20 and 65 years. For inclusion in the e-cigarette, cigarette smokers, and dual user groups, a self-reported questionnaire was conducted to ascertain a minimum daily use of e-cigarettes only or dual use for at least 1 year. Healthy non-smokers were checked for cotinine (a metabolite of nicotine) in urine samples to certify no use of nicotine products.

No participants had a history of allergy which was confirmed with a negative skin-prick test to a standard panel of common allergens (pollens, animal danders, and dust mites) as previously described [43]. None had a history of lung and airway disease, history of infection or exacerbation, or use of local or systemic anti-inflammatory drugs within 4 weeks prior to the examination, as assessed by questionnaire and confirmed by negative physical examination. All participants had normal lung function as any participants with forced expiratory volume in one second (FEV_1_)/vital capacity (VC) value ≤ 0.7 were excluded from the study.

### Measurements

All measurements were taken on a single occasion (one participant/day) in the following order due to logistic reasons.

#### Spirometry

Lung function test parameters (FEV_1_ and VC) were recorded by spirometry which was conducted using a wedge spirometer (Vitalograph, Buckingham, UK) according to American Thoracic Society/European Respiratory Society (ATS/ERS) recommendations [44]. Three measurements were taken for each parameter and the highest value was used as baseline. Local lung function reference values were used [45, 46].

#### Fractional nitric oxide in exhaled air (FeNO)

FeNO was measured by NIOX VERO (Circassia AB, Uppsala, Sweden) device following the manufacturer’s instructions and the ATS/ERS guideline [47]. Participants were instructed to rinse their mouth with water for 1 min before the measurements, exhale fully to empty their lungs, enclose the mouthpiece connected with a filter with lips to prevent air leakage, inhale deeply through the filter to fill in the lungs with maximum capacity and then exhale slowly through the filter into the device for 10 s at a constant flow rate of 50 ml/s. Two consecutive measurements were taken and the mean value was used for analysis.

#### Blood sampling

Peripheral blood was collected in three vacutainer tubes (BD vacutainer, Swemed). Blood in EDTA and in heparin was used for flow cytometry analysis. Blood with a clot activator was used to collect serum and stored at -80 ^0^C until further analysis.

#### Saliva sampling

Participants were instructed not to eat, drink, or perform oral hygiene 1.5 h before the visit. Before collection, participants were asked to swallow any saliva present in the mouth and look at lemons. Stimulated production of saliva was collected by spitting for 10 min in a falcon tube. The sample was weighed, aliquoted, and stored at -80 ^0^C until analysis was done.

#### Bronchial responsiveness

A bronchial challenge test was performed with methacholine bromide using a nebulizer with an output of 0.4–0.6 L/min as described previously [48, 49]. The participants inhaled methacholine 0.5–32 mg/mL in doubling doses. Inhalation was performed with tidal breathing for 1 min with 5 min interval between doses. FEV_1_ was measured 3 min after inhalation of each dose. The challenge test was stopped when a ≥ 20% reduction of FEV_1_ from baseline occurred or when the maximum dose was inhaled. The result is expressed as a cumulative provocation dose (PD20).

#### Sputum induction and sample processing

Sputum induction and sample processing were performed as previously described [43]. Following salbutamol (0.4 mg) inhalation, sputum was induced by inhalation of saline at increasing concentrations (0.9%, 3%, 4%, 5%) using an ultrasonic nebulizer (De Vibliss Ultraneb 2000) with an output of 3mL/min. Each concentration was inhaled for 7 min followed by a FEV_1_ measurement. Participants were then asked to rinse their mouth with water, sniff their nose, and cough deeply to expectorate sputum which was collected in a centrifuge tube. The sample was considered adequate when it was more than 2 g and macroscopically appeared to be saliva-free.

Sputum was processed within 2 h of collection. First, the sputum sample was treated with an equal volume of 0.1% dithiothreitol and kept in a shaking water bath for 15 min at 37 ^0^C temperature. Second, the sample was filtered through a nylon mesh (70 μm) to remove mucous and debris and centrifuged for 10 min (1.500×g). the supernatant was aliquoted and stored at -80 ^0^C until further analysis. The cell pellet was resuspended with 1 ml DPBS. Total cell counts and viability were performed with trypan blue staining. Sputum samples containing less than 30% squamous cells were considered successful and included in the analysis.

### Analysis

#### Flow cytometry

The total reactive oxygen species (ROS) in blood/ sputum cells were quantified with CellROX green reagent (ThermoFisher Scientific, Rockford, lL, US). The surface expression of Toll-like receptor (TLR)2 and TLR4 on blood/ sputum immune cells were identified with TLR monoclonal antibodies (anti-TLR2-BV421, Cat# 565350, anti-TLR4-BV711, Cat#564404, BD Bioscience, BD Pharmingen™, US).

Anti-CD3-Per cy5.5 or Anti-CD4-FITC (Cat# 560835, Cat# 555346, BD Bioscience, BD Pharmingen™, US) was used for gating T cells both in peripheral blood and induced sputum. The level of intracellular cytokines from these T cells were detected with anti-IL-2-FITC, anti-IL-4-APC, anti-IL-13-PE, and anti-IFN-r-PE (Cat# 3400448, Cat# 560671, Cat# 554571, Cat# 559326, BD Bioscience, BD Pharmingen™, US).

To analyze cell distribution in peripheral blood, anti-CD45-PE and anti-CD14-FITC (Cat# 555397, Cat# 555397, BD Bioscience, BD Pharmingen™, US) were used in TrueCOUNT™ (blood).

The flow cytometric acquisition was performed by LSR Fortessa™ (BD Bioscience, US). The data was analysed using FlowJo software (BD bioscience, US), and calculated as median fluorescence intensity (MFI) or positive cell percentage for intracellular cytokine staining.

#### Enzyme linked immunosorbent assay (ELISA)

The level of Interleukin (IL)6 (Cat#DY206), IL8 (Cat#DY208), MMP9 (Matrix Metallopeptidase 9; Cat#DY911) and TIMP1 (Tissue Inhibitor of Metalloproteinase 1; Cat#DY970) and CC16 (club cell protein 16; Cat#DY4218) was measured in serum, saliva and sputum supernatant. SLPI (Secretory Leukocyte Protease Inhibitor; Cat#DY1274-05), ELAFIN (Elastase-specific Inhibitor; Cat#DY1747) and IL13 (Cat#DY213) were measured in serum. Soluble (s) CD14 (Cat#DY383) and sTLR2 (Cat#DY2616) were measured in serum and sputum. All measurements were performed with ELISA Duoset kit (R&D, USA) following the manufacturer’s instructions.

### Statistics

The results are presented as medians with interquartile range or mean with standard deviation. The median comparison between groups was assessed by non-parametric Kruskal-Wallis signed rank test followed by Mann-Whitney test as a post hoc test. The mean comparison between groups (lung function) was assessed by ordinary one-way ANOVA. A *p*-value < 0.05 was considered significant. All data were analysed using GraphPad Prism 9 software.

## Results

### Demographics of the study population

Baseline characteristics of the study population are shown in Table 1. The median age of the participants was between 26 and 30 years in all groups. All participants with a baseline FEV_1_/VC ≥ 0.7 were included in the study. Participants reported to use e-cigarettes for a median of 2.7 (IQR 2–3) years. The median pack year for cigarette smokers was 4.9 (IQR 2.4–13) years in those who only smoked traditional cigarettes and 1.7 (IQR 0.9–2.7) years in dual users.

**Table 1:** Characteristics of the study participants

	Healthy non-smokers *N* = 22	Cigarette smokers *N* = 20	E-cigarette users *N* = 20	Dual users *N* = 20	*P* value
Age, years, median (IQR)	27 (24–36)	30 (24.5–39.5)	27 (24–29)	26 (23.5–28.5)	0.3
Sex, n, male/female	13/9	6/14	10/10	12/8	
BMI, kg/m2, median (IQR)	23.9 (22–27.4)	23.2 (21.2–28.3)	23.7 (20.8–26)	22.7 (21.3–26.9)	0.8
FEV_1_^#^, L, mean (SD)	4.1 (0.9)	3.3 (0.75) *	4.1 (0.9)	3.9 (1)	0.03
% pred FEV_1_^#^, mean (SD)	98.6 (12.2)	96.6 (10.8)	97.8 (27.3	96.8 (17.1)	0.98
VC^#^, L, mean (SD)	4.7 (1.1)	3.8 (0.9)	4.5 (1)	4.3 (1.1)	0.06
% pred VC^#^, mean (SD)	92.9 (10.8)	91 (12.6)	90.6 (12)	88.4 (14.1)	0.7
FEV_1_/VC^#^, %, mean (SD)	87 (5.8)	87.4 (9.1)	91.8 (7.4)	90.7 (5.8)	0.08
E-cigarette use, total years, median (IQR)	-	-	2.7 (2–3)	-	
Cigarette smoking, pack-years, median (IQR)	-	4.9 (2.4–13)	-	-	
Dual use, median (IQR), E-cigarette use, total years Cigarette smoking, pack-years	-	1.7 (0.9–2.7)	1.5 (1–2.2)		

IQR: Interquartile Range, BMI: Body Mass Index, FEV_1_: Forced Expiratory Volume in one second, VC: Vital Capacity, % pred: percentage predicted, SD: Standard Deviation. Statistical significance was tested by Kruskal-Wallis followed by the post hoc Mann-Whitney test. #statistical significance was tested by ordinary One-way ANOVA. * *p* < 0.05, compared to healthy non-smokers

### Effects on FeNO and bronchial responsiveness

**Fig. 1:**
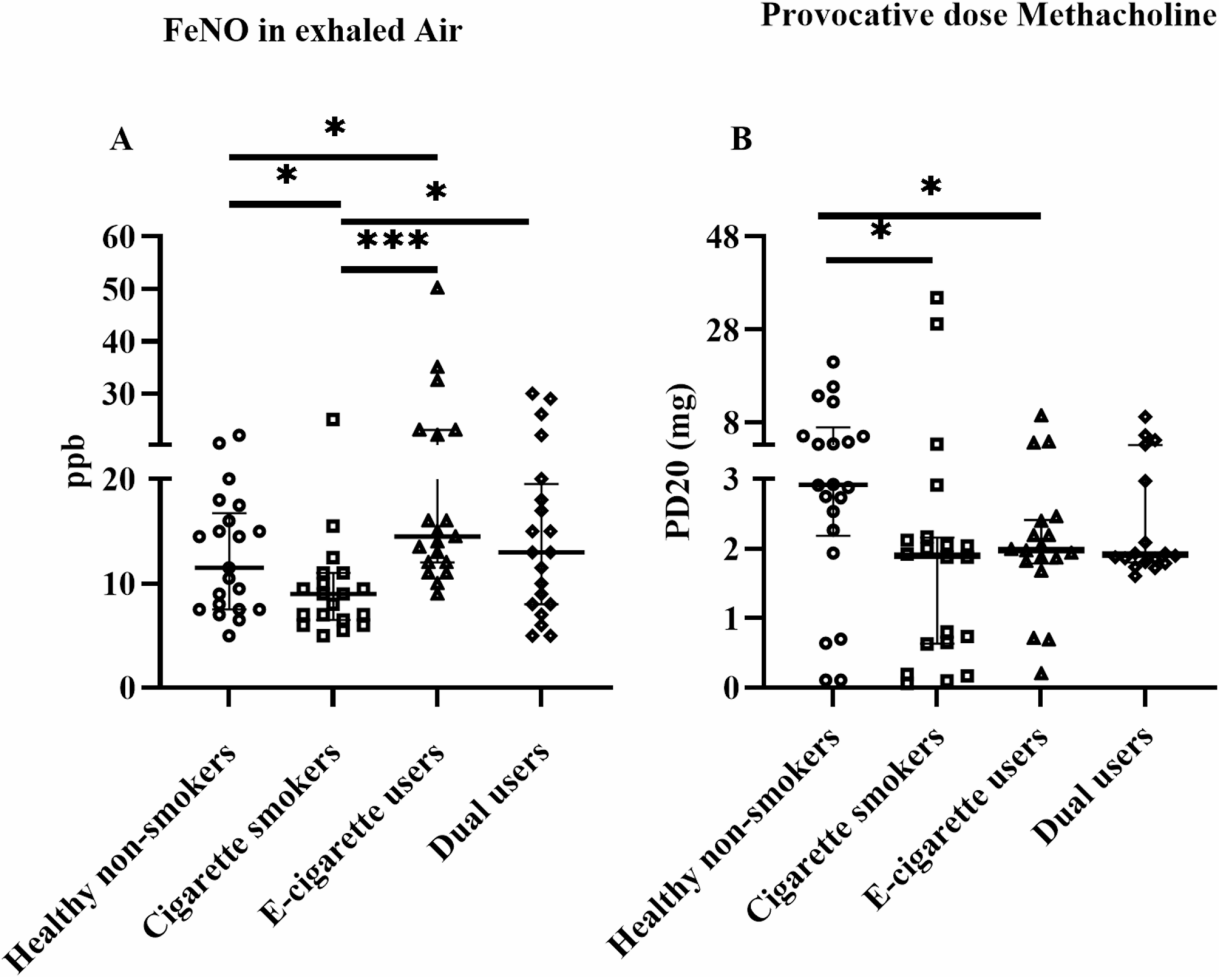
Fractional nitric oxide (FeNO) levels in exhaled air, measured by NIOX VERO (**A**). Cumulative Provocation dose of methacholine that produces a 20% drop of FEV_1_ (**B**). Data presented as median with interquartile range. Statistical significance was tested by Kruskal-Wallis followed by the post hoc Mann-Whitney test. * *p* < 0.05, ** *p* < 0.01, *** *p* < 0.001

Exhaled NO was significantly higher in e-cigarette users (14 ppb, *p* = 0.04) compared to healthy non-smokers (11 ppb) (Fig. 1A). In cigarette smokers, FeNO level was significantly lower (9 ppb, *p* = 0.04) compared to healthy non-smokers (Fig. 1A). A significantly higher FeNO was also found in e-cigarette users (*p* < 0.0001) and dual users (*p* = 0.03) compared to cigarette smokers (Fig. 1A). In addition, bronchial responsiveness increased significantly to methacholine at a lower dose in e-cigarette users (1.9 mg, *p* = 0.01) and cigarette smokers (1.9 mg, *p* = 0.01) compared to healthy non-smokers (2.9 mg) (Fig. 1B).

### Effects on oxidative stress in blood and induced sputum

**Fig. 2:**
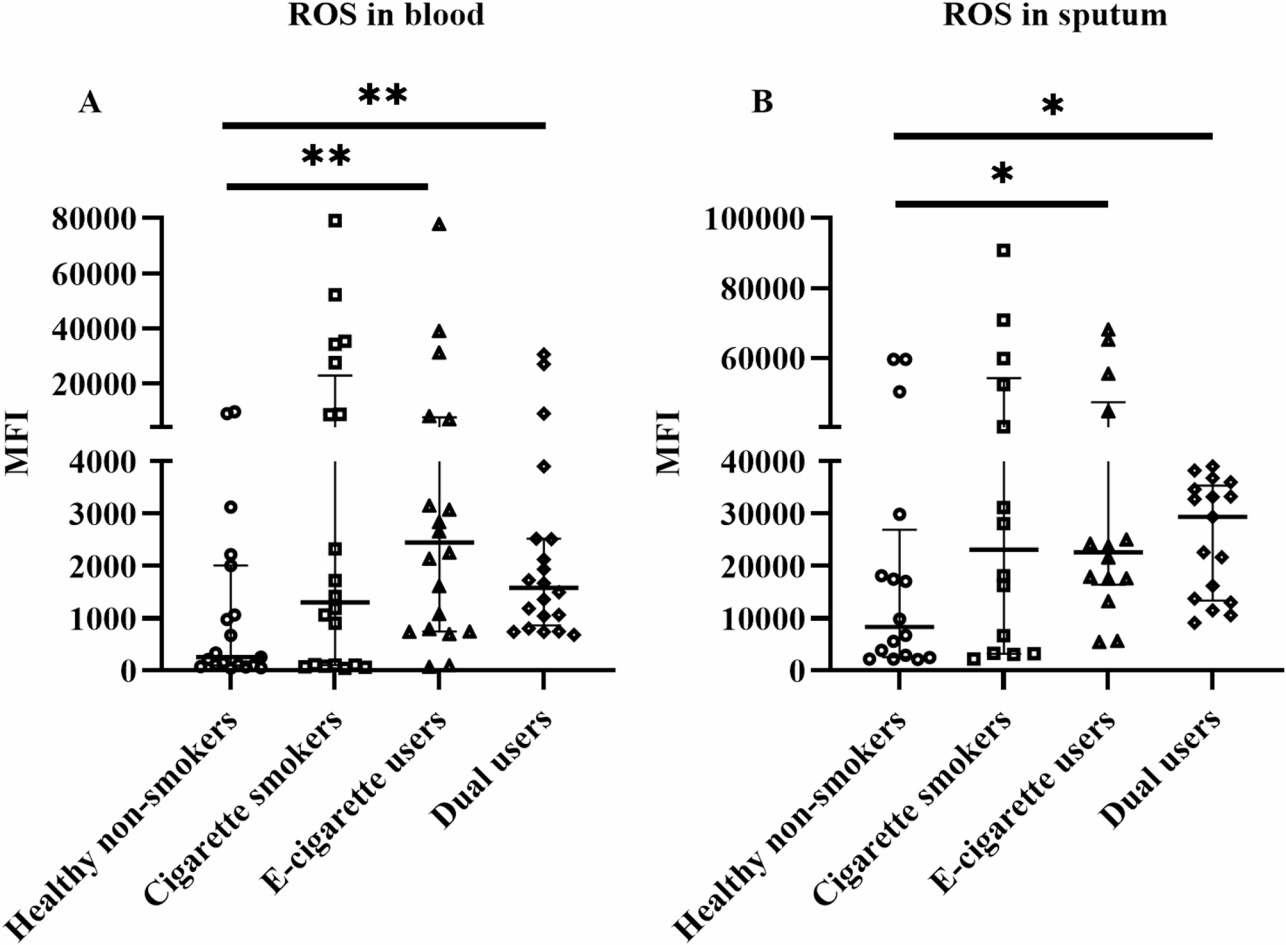
Total cellular reactive oxygen species (ROS) levels in the blood (**A**) and sputum (**B**), analyzed by flow cytometry. Data presented as median with interquartile range. Statistical significance was tested by Kruskal-Wallis followed by the post hoc Mann-Whitney test. * *p* < 0.05, ** *p* < 0.01

Oxidative stress was assessed by intracellular ROS production which was significantly higher in e-cigarette users (*p* = 0.005) and dual users (*p* = 0.003) compared to healthy non-smokers in blood (Fig. 2A). In sputum, ROS was also significantly higher in e-cigarette users (*p* = 0.04) and dual users (*p* = 0.04) compared to healthy non-smokers (Fig. 2B).

### Effects on differential cell counts in blood and immune cells in sputum

The concentrations of granulocytes did not differ significantly in any groups compared to healthy non-smokers (Additional file 1: Table S1). The concentration of lymphocytes was significantly higher in smokers (*p* = 0.03) and dual users (*p* = 0.003) compared to healthy non-smokers in blood (Additional file 1: Table S1). Total immune cell numbers were significantly higher in sputum in e-cigarette users compared to healthy non-smokers (Additional file 1: Figure S1).

### Effects on surface TLR2 and TLR4 expression in blood and induced sputum

**Fig. 3:**
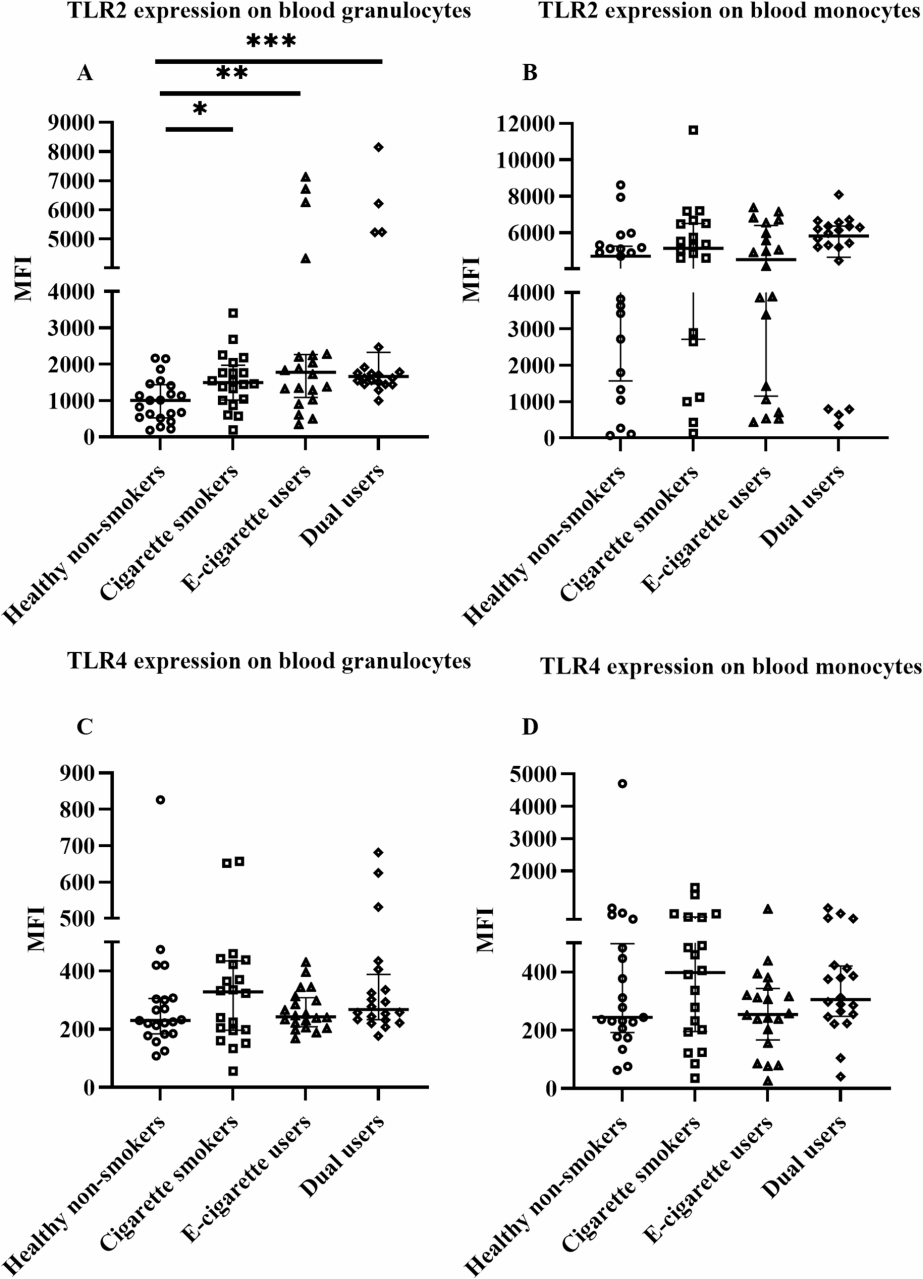
Surface expression of TLR2 (**A**– **B**) and TLR4 (**C**– **D**) on blood leucocytes, analyzed by flow cytometry. Data presented as median with interquartile range. Statistical significance was tested by Kruskal-Wallis followed by the post hoc Mann-Whitney test. * *p* < 0.05, ** *p* < 0.01, *** *p* < 0.001

**Fig. 4:**
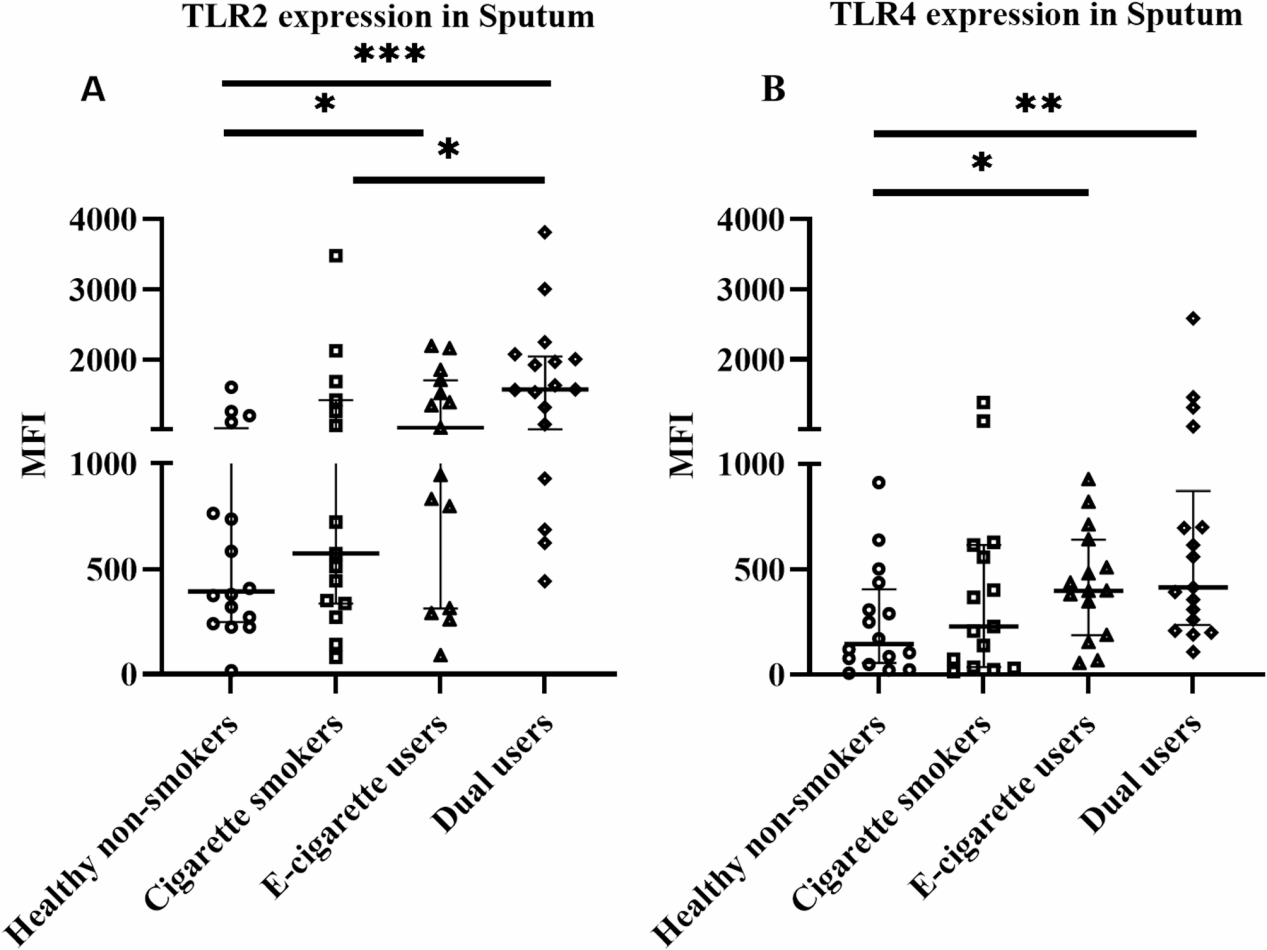
Surface expression of TLR2 (**A**) and TLR4 (**B**) in sputum immune cells, analyzed by flow cytometry. Data presented as median with interquartile range. Statistical significance was tested by Kruskal-Wallis followed by the post hoc Mann-Whitney test. * *p* < 0.05, ** *p* < 0.01, *** *p* < 0.001

In blood granulocytes, TLR2 expression was significantly higher in e-cigarette users (*p* = 0.005), cigarette smokers (*p* = 0.01), and dual users (*p* < 0.0001) compared to healthy non-smokers (Fig. 3A). In blood monocytes, TLR2 expression was not significantly altered in any group compared to healthy non-smokers (Fig. 3B). The expression of TLR4 did not differ in any group or cell type in blood (Fig. 3C and D). In sputum immune cells, both TLR2 and TLR4 expressions were significantly higher in e-cigarette users (*p* = 0.04 and *p* = 0.03) and dual users (*p* < 0.0001 and *p* = 0.004) compared to healthy non-smokers (Fig. 4A and B).

### Effects on sTLR2 and sCD14

**Fig. 5:**
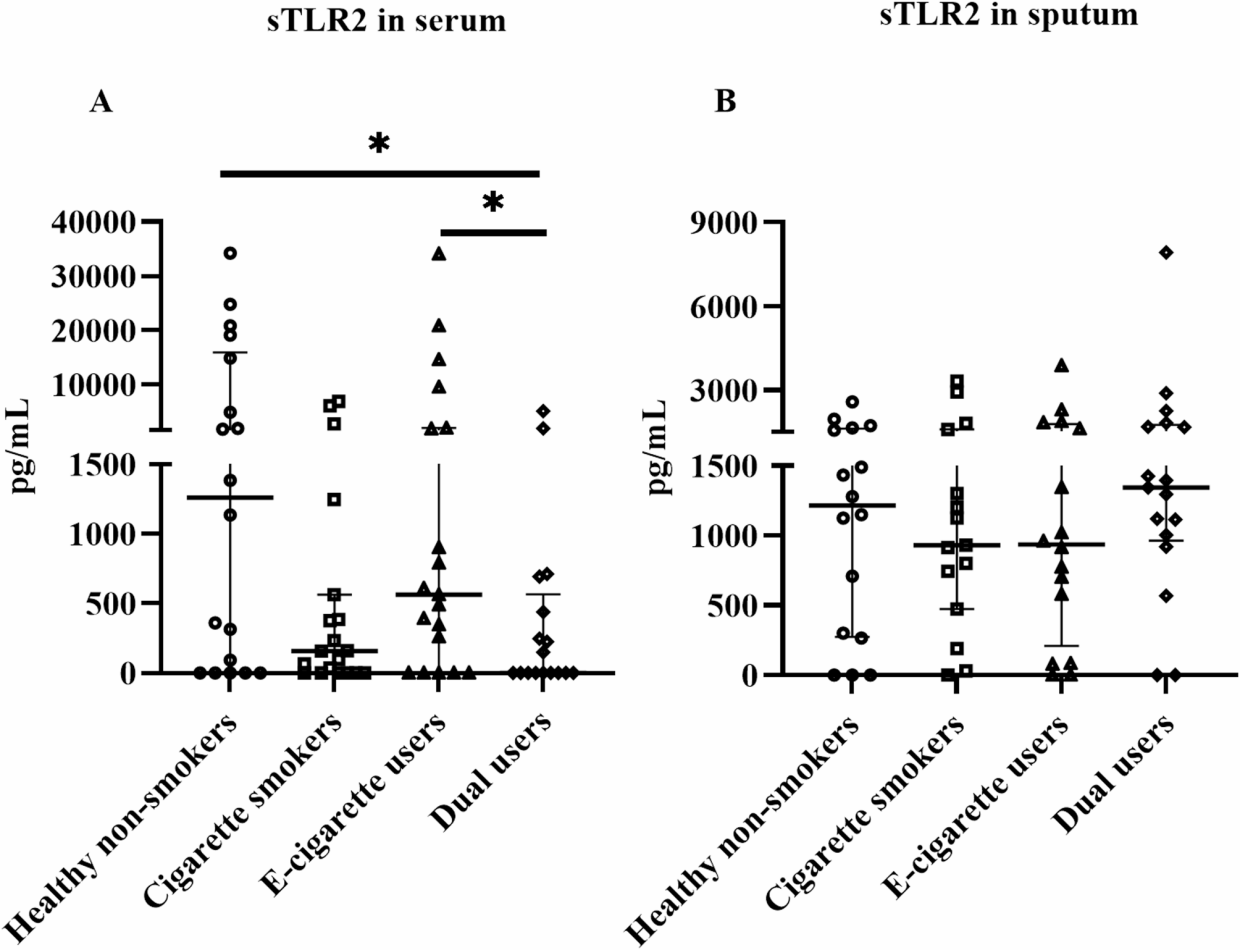
Soluble TLR2 in serum (**A**) and in sputum (**B**) supernatant measured by ELISA. Data presented as median with interquartile range. Statistical significance was tested by Kruskal-Wallis followed by the post hoc Mann-Whitney test. * *p* < 0.05

The level of sTLR2 in serum was significantly lower in dual users (*p* = 0.02) compared to healthy non-smokers (Fig. 5A). However, sTLR2 in sputum (Fig. 5B) and sCD14 in serum (Additional file 1: Figure S2A) and in sputum (Additional file 1: Figure S2B) did not change between groups.

### Effects on intracellular T cells cytokines in blood and sputum

**Fig. 6:**
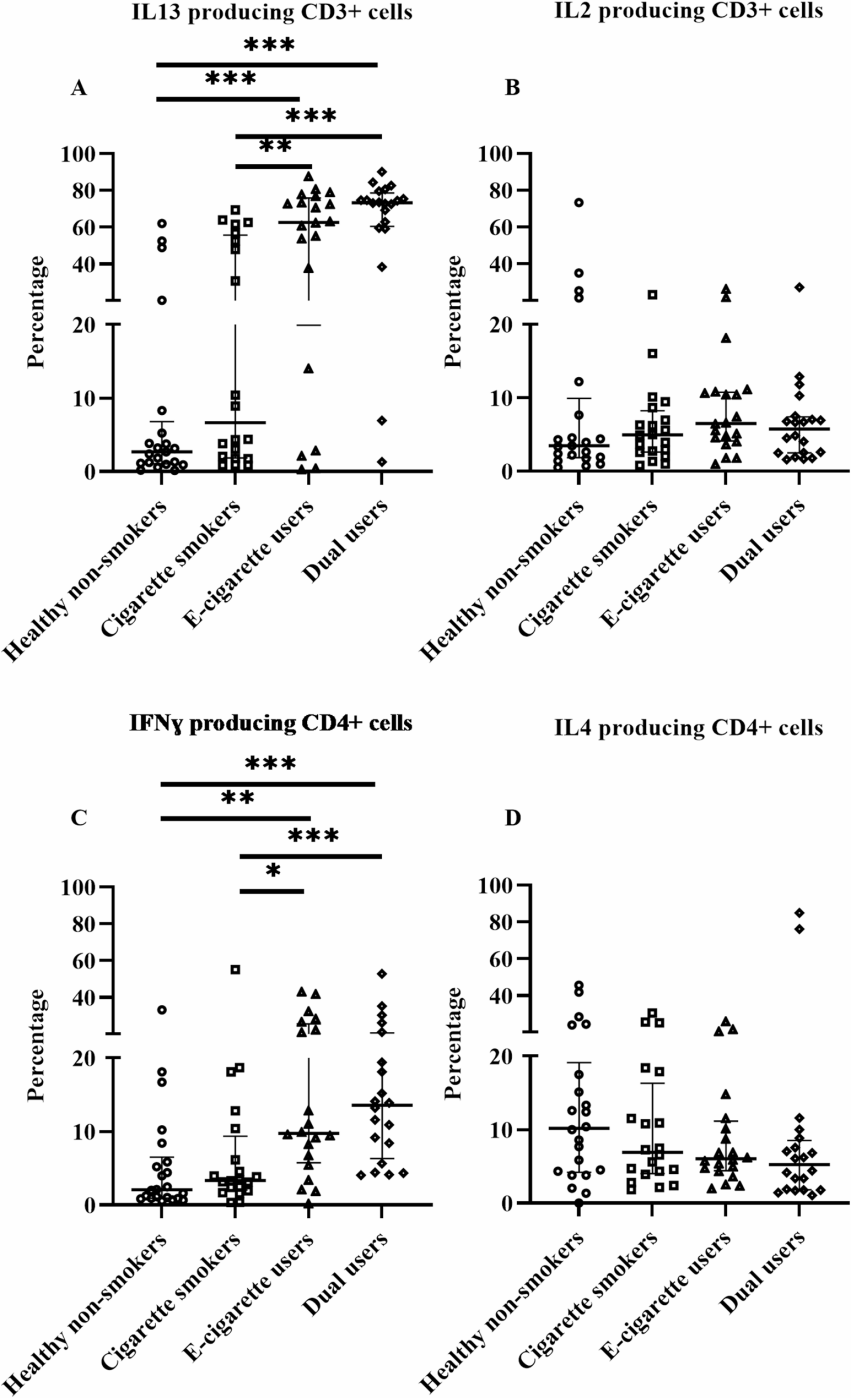
T cell cytokines profile in CD3 positive (**A**– **B**) and CD4 positive (**C**– **D**) T cells in blood, analyzed by flow cytometry. Data presented as median with interquartile range. Statistical significance was tested by Kruskal-Wallis followed by the post hoc Mann-Whitney test. * *p* < 0.05, ** *p* < 0.01, *** *p* < 0.001

In blood, the percentage of T cells that produce IL13 was significantly higher in e-cigarette users (*p* = 0.0001) and dual users (*p* < 0.0001) compared to healthy non-smokers (Fig. 6A). The percentage of IFNɣ producing T cells was also significantly higher in e-cigarette users (*p* = 0.001) and dual users (*p* < 0.0001) compared to healthy non-smokers (Fig. 6C). However, the percentage of IL2 or IL4-producing T cells did not differ in any exposure groups compared to healthy non-smokers (Fig. 6B and D). In sputum, the percentage of cytokine-producing T cells were not significantly different in any of the groups compared to healthy non-smokers (Additional file 1: Figure S3A– S3D).

### Effects on extracellular cytokines in serum, sputum, and saliva

**Fig. 7:**
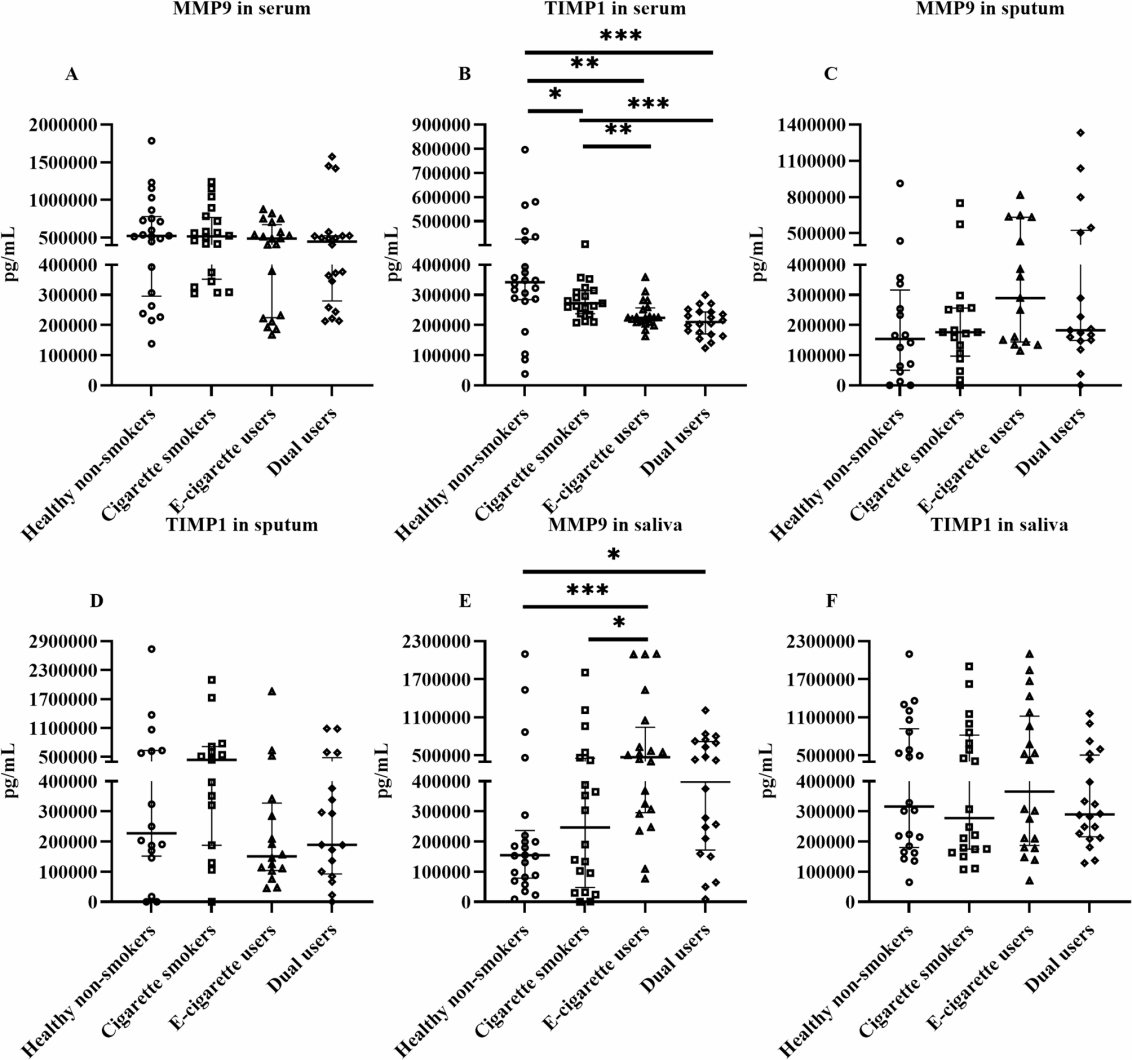
The concentration of tissue injury/repair markers in serum, sputum, and saliva (**A**– **F**), measured by ELISA. Data presented as median with interquartile range. Statistical significance was tested by Kruskal-Wallis followed by the post hoc Mann-Whitney test. * *p* < 0.05, ** *p* < 0.01, *** *p* < 0.001

In serum, MMP9 was not significantly different in any group. On the other hand, TIMP1 was significantly lower in e-cigarette users (*p* = 0.001), cigarette smokers (*p* = 0.03), and dual users (*p* = 0.0002) compared to healthy non-smokers (Fig. 7A and B). In addition, TIMP1 was significantly decreased in e-cigarette users (*p* = 0.004) and dual users (*p* = 0.0001) compared to cigarette smokers (Fig. 7B). However, SLPI, ELAFIN, CC16, and IL13 were not significantly different in any groups in serum (Additional file 1: Figure S4A– S4D). In sputum, none of the investigated markers were significantly different in any groups compared to healthy non-smokers (Fig. 7C and D and Additional file 1: Figure S4E– S4F). In saliva, MMP9 was significantly higher in e-cigarette users (*p* = 0.0005) and dual users (*p* = 0.03) compared to healthy non-smokers (Fig. 7E). However, no other cytokines showed any significant change in saliva (Fig. 7F and Additional file 1: Figure S4G– H). Furthermore, IL6 and IL8 were undetectable in serum, and IL6 was undetectable in sputum and saliva.

## Discussion

In this study, we investigated the local and systemic effects of e-cigarette use compared with cigarette smokers, dual users, and healthy non-smokers. Our results demonstrated that FeNO level was higher in e-cigarette users and lower in cigarette smokers, and airway hyperresponsiveness was increased both in e-cigarette users and in cigarette smokers compared to healthy non-smokers. Our results also demonstrated that ROS level was higher in e-cigarette users and dual users in blood and sputum compared to healthy non-smokers. While TLR2 was increased in all groups in blood granulocytes, sTLR2 was decreased in dual users in serum. Both TLR2 and TLR4 were increased in e-cigarette users and dual users in sputum compared with healthy non-smokers. In addition, the percentage of IL13 and IFNɣ-producing T cells was increased in e-cigarette users and dual users, TIMP1 was decreased in serum and MMP9 was increased in saliva in both e-cigarette users and dual users compared to healthy non-smokers.

FeNO is an important non-invasive biomarker for the assessment of Type-II inflammation in asthma, where FeNO levels usually are elevated [50, 51]. In our study, all participants had a negative skin allergy test for common allergens. FeNO levels in COPD are reported to be inconsistent since it is more related to smoking habits and disease severity [52, 53]. It is well-known that decreased FeNO levels are associated with cigarette smoking [9, 54]. Our results also showed a significant reduction of FeNO levels in cigarette smokers compared to healthy non-smokers. On the contrary, significantly increased FeNO levels were observed in e-cigarette users compared to healthy non-smokers. It was reported in a previous cross-sectional study that FeNO levels increased after acute e-cigarette exposures independent of nicotine content [27]. In another study, FeNO levels increased in participants who were exposed to nicotine-containing e-cigarettes for a week [55]. However, other studies found decreased or unaltered FeNO levels in e-cigarette users compared to controls [24, 56]. Under normal conditions, NO is primarily produced in bronchial epithelium by inducible nitric oxide synthase (iNOS) enzyme-mediated by interferon-gamma (IFNγ). During type 2 inflammation, overexpression of type 2 inflammatory cytokines such as IL4 and IL13 leads to overproduction of NO by overexpressing iNOS [57, 58]. Cigarette smoke contains a very high level of NO and therefore FeNO reduction could be the result of feedback inhibition [54]. However, e-cigarettes may exert their effects on FeNO in the lungs in ways that might be different and opposite from what is known to be caused by traditional cigarettes. Therefore, follow-up studies are needed to fully understand how e-cigarettes alter FeNO levels and to shed light on the inconsistent results found in the previously published papers.

Bronchial hyperresponsiveness is an important characteristic of asthma. However, it can also be a predictor in the development of COPD [59, 60]. Previous studies have shown that cigarette smoking is associated with bronchial hyperresponsiveness [61–63]. Some studies have also found an association between e-cigarette use and increased bronchial responsiveness [64, 65]. Similar to these findings, our results showed that bronchial responsiveness was increased in both e-cigarette users and cigarette smokers compared to healthy non-smokers. This might be an indication of ongoing inflammation which in the long run might contribute to the development of airway diseases.

Oxidative stress induced by the production of ROS plays an important role in the pathogenesis of chronic pulmonary diseases such as asthma, COPD, fibrosis, and lung cancer [66, 67]. It is well-established that cigarette smoke-induced oxidative stress is a key molecular event in the pathogenesis of smoking-related diseases including COPD [68, 69]. Several studies have already found evidence of e-cigarette-induced ROS generation and its potential role in the development of pulmonary toxicity [70–72]. In the present study, ROS was significantly increased in e-cigarette users compared to healthy non-smokers in both blood and sputum. It was also increased in dual users compared to healthy non-smokers. E-cigarettes seem to play a major role in the observed effects in dual users since there was no significant change in ROS level in cigarette smokers compared to healthy non-smokers in our study.

Variations in the white blood cells/leukocytes have been found to be caused by inflammation in the lungs [73]. Increased neutrophils are associated with COPD and increased eosinophil numbers are associated with asthma [73]. Cigarette smoking is also associated with increased leukocytes including lymphocytosis and neutrophilia [74]. In our study, granulocytes in blood were higher in e-cigarette users and cigarette smokers, although not significant. However, we have found significantly increased lymphocyte counts in blood in smokers compared to non-smokers. Increased lymphocytes were also significantly associated with dual users. Since we have not observed any significant change in e-cigarette users, cigarette smoking may have dominated the outcome in dual users.

Toll-like receptors (TLR) are an important part of the pattern recognition receptor (PRR) family. Among other TLR found in humans, TLR4 and TLR2 recognize pathogen-associated molecular patterns (PAMPs) such as lipopolysaccharide and lipoprotein, respectively, and get activated [75]. The role of a co-receptor CD14 is also critically important in TLR signalling especially TLR4 [76, 77]. Activated TLR induces the release of inflammatory cytokines and thereby plays a vital role in mediating innate immune response [78]. TLR2 and TLR4 are mostly expressed by immune cells and lung epithelial cells and have major implications in the pathogenesis of chronic lung diseases such as COPD and asthma [78–82]. Previous studies have found that TLR expression is induced by cigarette smoking [79, 80] and their level is upregulated in COPD patients [78, 79, 81]. For example, an increased level of TLR2 in blood monocytes was found in a study in COPD patients [83]. On the contrary, reduced TLR2 expression was found in sputum neutrophil and alveolar macrophage in smokers with COPD [84, 85]. In addition, TLR2 and TLR4 expression were found to be upregulated in neutrophilic asthma [86]. In our study, only TLR2 was significantly increased in e-cigarette users and dual users in blood granulocytes compared to healthy non-smokers. Whereas, both TLR2 and TLR4 were significantly increased in e-cigarette users and dual users in sputum cells compared to healthy non-smokers. E-cigarettes seemed to play a major role in the observed TLR effects in dual users in our study. In addition, sTLR2 and sCD14 are considered decoy receptors, that negatively regulate TLR2 signalling and cytokine release [87]. In our study, we found a significantly reduced level of sTLR2 in dual users in serum compared to healthy non-smokers. Increased TLR2 and decreased sTLR2 might indicate disruption of the negative regulatory mechanism. However, no negative correlation was found (data not shown).

Cytokines play a major role in maintaining lung homeostasis and in the pathogenesis of chronic lung diseases like asthma and COPD [88]. Major cytokines involved in asthma include IL4, IL5, and IL13 derived from CD4^+^ T cells type II [88, 89]. Smokers without COPD have been shown to have an increased proportion of cells producing both IL13 and IL4 [90]. Also, an increased proportion of cells expressing IL4 and IL13 has been found in smokers with chronic bronchitis compared to asymptomatic smokers [91]. IL13 induces goblet cell metaplasia, hyperplasia, and MUC5AC production in Normal Human Bronchial Epithelial (NHBE) cells [92, 93]. Major proinflammatory and T-cell cytokines involved in COPD includes TNFα, IL1β, IL6, IL8, IL2 and IFNɣ [88, 94] where pro-inflammatory cytokines are released by the NF-κB pathway and T cell cytokines IL2 and IFNɣ derive from CD4^+^ T cells type II [94, 95]. Our results showed that the percentage of T cells producing IL13 and IFNɣ increased significantly in e-cigarette users and dual users in blood compared to healthy non-smokers as well as compared to cigarette smokers. In our previously published study, the prevalence of cough and mucous production was found to be significantly higher among e-cigarette users compared to non-smokers [13]. In addition, our observed increased levels of FeNO in e-cigarette users could be mediated by this increased production of IL13.

Besides cytokines, tissue injury marker MMP9 and tissue injury repair marker TIMP1 are also associated with COPD [94]. MMP9 can be induced by TNFα and TMP1 by IL10 in COPD. Cigarette smoking can also increase the release of MMP9 and TIMP1 [94, 95]. An increased level of MMP9 was found in serum in smokers with COPD compared to non-smokers [96]. Contrary to this, our results did not show any significant change in MMP9 in any group, and we detected significantly reduced TIMP1 in smokers compared to healthy non-smokers in serum. Significantly reduced TIMP1 level was also detected in e-cigarette users and dual users compared to both healthy non-smokers and cigarette smokers in serum. This might indicate a significant downregulation of TIMP1 and, thereby, impairment in tissue repair mechanism. Our results, on the other hand, found an increased MMP9 level in e-cigarette users and dual users in saliva compared to healthy non-smokers.

A major strength of the present study is that the study groups were matched with age and BMI. Any participants with reduced lung function (FEV_1_/VC ratio ≤ 0.7) at baseline, positive skin allergy test, no vaccination against COVID-19, history/sign of respiratory symptoms/high blood pressure/other diseases were excluded from participation. Although the small sample size of the study may limit the power of the investigated outcome, strict inclusion criteria increase the validity of the exposure outcome. This study also examines new measures of dual use of e-cigarettes and traditional cigarettes on local and systemic outcomes. Assessments of exposure to e-cigarettes and traditional cigarettes were based on self-reported questionnaires. The extent to which they capture the true exposure is not validated. However, the high validity of self-reported tobacco use with urinary cotinine level assessment among young adults in Sweden was found in our recently published paper [97]. Another strength is that e-cigarette use was assessed not just based on a yes/no questionnaire but from the information on the duration and frequency of e-cigarette use, type of the device, presence or absence of nicotine or/and flavours in the e-liquid. However, a detailed assessment was not possible due to wide variation in the frequency of use, type of the device, and e-liquid. Future studies with detailed puff topography and serum cotinine as a nicotine exposure biomarker, involving a larger population, would provide better insights into individual use patterns and dose-response relationships. In addition, it was not possible to gate the neutrophils, eosinophils, and basophils in the blood and sputum due to technical issues.

## Conclusion

In this study, increased baseline FeNO and bronchial responsiveness were observed in e-cigarette users compared to healthy non-smokers. Oxidative stress markers, surface TLR expressions, and the proportion of inflammatory cytokines IL13 and IFNɣ-producing cells were also higher in e-cigarette users as well as in dual users. Our results indicate that inflammatory response and innate immune receptors were altered both locally and systemically in e-cigarette users as well as in dual users. It should be noted that these changes were observed among e-cigarette users although they have not used e-cigarettes for more than 1–3 years.

## Electronic supplementary material

Below is the link to the electronic supplementary material.

**Supplementary Material 1**: **Additional file 1**

## Data Availability

The datasets used and/or analysed during the current study are available from the corresponding author on reasonable request.

## Abbreviations

COPD: Chronic obstructive pulmonary disease
CC16: Club cell protein 16
CD: Cluster differentiation
Dual Use: Combined use of e-cigarettes and traditional cigarettes
e-cigarette: E/electronic cigarette
ELAFIN: Elastase-specific Inhibitor
EDTA: Ethylenediaminetetraacetic acid
FEV1: Forced expiratory volume in one second
FeNO: Fractional Exhaled Nitric Oxide
IL: Interleukin
IFN: Interferon
iNOS: Inducible nitric oxide synthase
MMP9: Matrix metallopeptidase 9
MFI: Median fluorescence intensity
MUC5AC: Mucin-5AC
NF-κB: Nuclear factor kappa B
PD: Provocation dose
PRR: Pattern recognition receptor
PAMP: Pathogen-associated molecular patterns
ROS: Reactive oxygen species
SLPI: Secretory leukocyte protease inhibitor
TLR: Toll-like receptor
TIMP: Tissue inhibitor of metalloproteinase
TNF: Tumour necrosis factor
VC: Vital capacity
