# Supplementary material for: Local and systemic effects in e-cigarette users compared to cigarette smokers, dual users, and non-smokers
Source: Respir Res. 2025 Jun 4;26:207. doi: 10.1186/s12931-025-03289-4 (PMC12139149; doi:10.1186/s12931-025-03289-4)
Supplement: Supplementary file 1 — Supplementary Material 1: Additional file 1 [file 12931_2025_3289_MOESM1_ESM.docx]

**Supplementary data**

**Table S1.** Differential cells count in blood

| **Blood (10^3^ cells/ml)** | **Healthy non-smokers**  **N=22** | **Cigarette smokers**  **N=20** | **E-cigarette users**  **N=20** | **Dual users**  **N=20** |
| --- | --- | --- | --- | --- |
| **Granulocytes** | 3572 (2637 – 4312) | 4344 (2979 – 5278) | 4276 (2876 – 5183) | 3789 (3550 – 5534) |
| **Lymphocytes** | 1861 (1539 – 2298) | 2226 (1966 – 2853) * | 2150 (1839 – 2586) | 2528 (2133 – 3371) ** |
| **Monocytes** | 369 (281 – 434) | 374 (305 – 589) | 392 (328 – 566) | 545 (305 – 795) |

Data presented as median with interquartile range. Statistical significance was tested by Kruskal-Wallis followed by the post hoc Mann-Whitney test. * p<0.05, ** p<0.01, compared to healthy non-smokers.

**Figure S1.** Total immune cells in sputum. Data presented as median with interquartile range. Statistical significance was tested by Kruskal-Wallis followed by the post hoc Mann-Whitney test.

**Figure S2.** Soluble CD14 in serum (A) and sputum (B) supernatant measured by ELISA. Data presented as median with interquartile range. Statistical significance was tested by Kruskal-Wallis followed by the post hoc Mann-Whitney test.

**Figure S3.** T cell cytokines profile in CD3 (A – B) and CD4 (C – D) positive T cells in sputum, analyzed by flow cytometry. Data presented as median with interquartile range. Statistical significance was tested by Kruskal-Wallis followed by the post hoc Mann-Whitney test.

**Figure S4.** The level of inflammatory biomarkers in serum (A – D), sputum (E – F), and saliva (G – H), measured by ELISA**.** Data presented as median with interquartile range. Statistical significance was tested by Kruskal-Wallis followed by the post hoc Mann-Whitney test.
